# A 3D Radiomics-Based Artificial Neural Network Model for Benign Versus Malignant Vertebral Compression Fracture Classification in MRI

**DOI:** 10.1007/s10278-023-00847-4

**Published:** 2023-05-30

**Authors:** Natália S. Chiari-Correia, Marcello H. Nogueira-Barbosa, Rodolfo Dias Chiari-Correia, Paulo M. Azevedo-Marques

**Affiliations:** 1 Medical Artificial Intelligence Laboratory of the Ribeirão, Preto Medical School, University of São Paulo, 3900 Bandeirantes Avenue, Ribeirão Preto, SP 14049-900 Brazil; 2 Department of Medical Imaging, Hematology and Oncology of the Ribeirão Preto Medical School, University of São Paulo, Ribeirão Preto, SP Brazil; 3 Department of Orthopedic Surgery, University of Missouri Health Care, Columbia, MO USA; 4 Department of Physics, Faculty of Philosophy, Sciences and Letters, University of São Paulo, Ribeirão Preto, SP Brazil

**Keywords:** Spine, Compression fractures, Magnetic resonance image, Medical image processing, Neural network models

## Abstract

To train an artificial neural network model using 3D radiomic features to differentiate benign from malignant vertebral compression fractures (VCFs) on MRI. This retrospective study analyzed sagittal T1-weighted lumbar spine MRIs from 91 patients (average age of 64.24 ± 11.75 years) diagnosed with benign or malignant VCFs from 2010 to 2019, of them 47 (51.6%) had benign VCFs and 44 (48.4%) had malignant VCFs. The lumbar fractures were three-dimensionally segmented and had their radiomic features extracted and selected with the wrapper method. The training set consisted of 100 fractured vertebral bodies from 61 patients (average age of 63.2 ± 12.5 years), and the test set was comprised of 30 fractured vertebral bodies from 30 patients (average age of 66.4 ± 9.9 years). Classification was performed with the multilayer perceptron neural network with a back-propagation algorithm. To validate the model, the tenfold cross-validation technique and an independent test set (holdout) were used. The performance of the model was evaluated using the average with a 95% confidence interval for the ROC AUC, accuracy, sensitivity, and specificity (considering the threshold = 0.5). In the internal validation test, the best model reached a ROC AUC of 0.98, an accuracy of 95% (95/100), a sensitivity of 93.5% (43/46), and specificity of 96.3% (52/54). In the validation with independent test set, the model achieved a ROC AUC of 0.97, an accuracy of 93.3% (28/30), a sensitivity of 93.3% (14/15), and a specificity of 93.3% (14/15). The model proposed in this study using radiomic features could differentiate benign from malignant vertebral compression fractures with excellent performance and is promising as an aid to radiologists in the characterization of VCFs.

**Supplementary Information:**

The online version contains supplementary material available at 10.1007/s10278-023-00847-4.

## Introduction

Vertebral compression fractures (VCFs) occur in different clinical scenarios, such as trauma, osteoporosis, and neoplastic infiltration [1]. In the case of a nontraumatic VCF, the diagnosis of the underlying disease may be challenging, especially in the elderly population [2]. The most frequent causes of benign and malignant nontraumatic VCFs are osteoporotic bone fragility and bone metastasis, respectively.

The study of benign and malignant VCF differentiation has been the subject of research for more than 20 years, making it possible to expand the understanding of imaging patterns that could help discriminate between these two conditions [2–4]. MRI has been established as the most relevant medical imaging technique for diagnosing malignant spinal disease, mainly because of its high sensitivity to bone marrow abnormalities [5, 6]. The criteria used to distinguish between benign and malignant VCFs in the clinical routine are related to vertebral body morphology and the signal intensity abnormalities of the bone marrow mainly observed on T1WI and T2WI [7, 8]. Some characteristics commonly associated with malignant fractures are i) metastasis in other vertebrae, ii) presence of an epidural or paravertebral soft-tissue mass, iii) abnormal signal of the pedicle or other posterior elements, iv) total replacement of bone marrow fat signal by low-signal tissue on T1-weighted sequence, and v) diffuse posterior vertebral border convexity. Concerning benign fractures, some characteristics frequently associated with them are i) additional benign fractures, ii) pedicle and posterior vertebral element with normal signal, iii) focal posterior vertebral border convexity, and iv) linear horizontal hypointense T1/T2 band. However, none of the above qualitative criteria are pathognomonic when used alone [3, 9].

In a recent study to evaluate the interobserver concordance and diagnostic accuracy to differentiate benign versus malignant VCF, Arana et al. concluded that these metrics are moderate at best, irrespective of medical or surgical specialty, years of clinical experience, or hospital type. In addition, they highlighted this result casts doubt on the reliability of using MRI findings together with clinical history as the basis for distinguishing benign from malignant VFC in routine clinical practice or multicenter studies [10]. Biopsies are also an important reference for the differential diagnosis between malignant and benign lesions; however, as it is an invasive technique, it should not be used as a standard method in clinical routine. In addition, there is already an understanding that, due to the spatial and temporal heterogeneity of tumors, other types of descriptors need to be used to allow a more complete characterization of the lesion [11, 12].

In the last decade, the role of medical imaging as a diagnostic tool has expanded, allowing it to become a potential critical pillar in the context of personalized medicine. This only became possible when medical images started to be treated as quantitative data [13]. Radiomics is a relatively recent approach with promising results, mainly in oncology, and it consists of the massive extraction of many quantitative features from medical images that can be correlated with clinical outcomes [11].

Previous studies used quantitative features extracted from MRI to differentiate benign from malignant VCFs. Frighetto-Pereira et al. [14] extracted 17 features to evaluate 103 VCFs (from 61 patients) using k-NN. Azevedo-Marques et al. [15] extracted 19 features derived from Fourier and wavelet transforms to classify 73 VCFs (54 benign and 19 malignant) from 47 patients. Frighetto-Pereira et al. [16] extracted 27 quantitative features to evaluate 102 VCFs using different classifiers from those used in their previous work. Casti et al. [17] proposed to segment the fractures using nine different techniques and extract 15 shape-based features from each segmentation result. Finally, Arpitha and Rangarajan [18] proposed a segmentation and classification method based on shape and texture to differentiate benign from malignant VCFs in 62 patients. They used the same set of features used in [14] and [16]. Although these studies have shown promising results, all of them had an exploratory goal and did not analyze the data to the point of proposing a radiomic signature capable of performing the differentiation between benign and malignant VCFs with suitable reliability. In addition, as above-mentioned, radiomics implies a "massive extraction" of features to obtain the maximum information. In previous studies, the largest number of extracted features was 27, which may lead to a lack of relevant information. Finally, all the studies only evaluated the central slice of T1-weighted sagittal MRIs. To the best of our knowledge, this is the first work that used a three-dimensional approach, which allows us to extract more information from the images [19].

Our hypothesis is that the massive and three-dimensional extraction of features from spinal MRIs may result in a radiomic signature capable of differentiating benign and malignant VCFs. The aim of this study was to distinguish between benign and malignant VCFs using an artificial neural network based on three-dimensionally extracted radiomic features from MRIs.

## Material and Methods

### Study Design

This retrospective study proposed the creation of a classification model for the differential diagnosis of benign and malignant vertebral compression fractures using quantitative features three-dimensionally extracted from MRIs.

### Dataset

This study was performed in compliance with Health Insurance Portability and Accountability Act regulations and was approved by the Institutional Review Board. Informed consent was waived.

The dataset of this study comprised sagittal T1-weighted lumbar spine MRIs obtained from consecutive patients diagnosed with benign or malignant vertebral compression fractures at the University Hospital. The MRIs were performed in the clinical setting using the Philips Achieva 1.5 T and 3 T MRI systems (Philips Medical Systems, Best, The Netherlands) and were retrieved from Picture Archiving and Communication System (PACS) in the Digital Imaging and Communications in Medicine (DICOM) format. The sagittal T1-weighted MRI sequences had the following parameters: 400–675.38/7–14 [TR (msec)/TE (msec)], 183.6–418.6/183.6–630.2 [FOVx (mm)/FOVy (mm)], 320–1750/320–1024 [Rows/Columns], 2–8 [Number of Averages], 160–767 [Pixel Bandwidth (hz/pixel)], 3–5 [Slice Thickness (mm)], 3–5.5 [Spacing between Slices (mm)] and 12–15 [Number of Slices]. The table containing the parameters for each exam can be seen in the electronic supplementary material.

To search the patients, a query on the RIS was performed from 2010 to 2019. Due to the system's limitation, it was only possible to select the search period, insert the anatomical region of the MRI (spine), and input the term "fracture" to filter the reports. Therefore, many cases in which the physician reported "absence of fracture" were also recovered. From the list generated by the RIS, the duplicated patients were eliminated, remaining in the data set only the oldest acquisition of the patient. This strategy was adopted to reduce the chances that the patient had started some treatment. After discarding duplicate patients and exams that did not have lumbar fractures, 375 cases that met the inclusion criterion, which was "patient with at least one vertebral fracture in the lumbar region" were selected. The exclusion criteria were (a) treatment (such as chemotherapy, radiotherapy, and surgery) before the MRI, (b) a fracture of traumatic etiology, (c) diagnosis not confirmed by biopsy (for the malignant fractures), (d) an old fracture and (e) patients under 18 years old (considering the MRI date).

To meet the exclusion criteria and define the ground truth, a rereading of all MRI examinations by a senior radiologist (blinded) with 20 years of experience in musculoskeletal radiology was performed, as well as the review of all available data in the medical records of the hospital information system (HIS), including biopsies, clinical and imaging follow-up. The labeling and exclusion of cases were carried out during the same process of checking the medical records. Regarding the malignant fractures, the spine biopsy was the main criterion to confirm the diagnosis and label the fracture, so patients who did not undergo this examination were excluded. As an additional evaluation, the review of the patient records to check the neoplasia follow-up was performed, and those who started treatment before the MRI were also excluded. Concerning the benign fractures, DEXA scan indicating the presence of osteoporosis and the rereading of the MRIs were used to confirm the diagnosis and label these fractures. As a complementary evaluation, an imaging and clinical follow-up of two years were performed to ensure that the group of fractures labeled as benign did not contain any vertebra with neoplastic infiltration imperceptible by the radiologist, which may occur, for example, in patients with early-stage multiple myeloma.

The study group comprised 91 patients (36 men and 55 women, with an average age of 64.24 ± 11.75 years) with 146 fractured vertebrae. Of these 91 patients, 47 (51.6%) had benign VCFs and 44 (48.4%) had malignant VCFs. In addition, 61 (67%) had only one fracture, 16 (17.6%) had two fractures, 7 (7.7%) had three fractures, 3 (3.3%) had four fractures, and 4 (4.4%) had five fractures.

Once the final list of patients has been established, the MRIs were retrieved from the PACS of the institution. For this, a Python script was developed to automatically download the exams using the following DICOM header information: PatientID, Modality, and StudyDate. In this script, an anonymization function was inserted for some information in the DICOM header: PatientName, InstitutionAddress, InstitutionName, OperatorsName, ProtocolName, RequestingPhysician, RequestedPROCedureDescription, and BodyPartExamined. This step ensured that all exams used in this work were adequately anonymized.

The 91 patients were split into training and test sets. The last 15 patients with benign fractures and the last 15 patients with malignant fractures retrieved from PACS were chosen as the test set. To avoid bias during test set evaluation, just one vertebral body per patient was used. To choose which VCF would be used for the cases where a patient had more than one fractured vertebra, the Python function *random.choice* was used, so that among the 46 vertebral bodies (from 30 patients chosen as the test set), 30 were used to test the model, and 16 were discarded. The 100 fractured vertebral bodies (54 benign and 46 malignant) from the remaining 61 patients were used to train the model.

### Preprocessing and Segmentation

Preprocessing and segmentation were performed by using 3D Slicer version 4.8.1 (https://www.slicer.org/). Linear transformation was applied to the images to rescale the intensity levels between 0 and 255 by using the *RescaleIntensityImageFilter* tool, which is included in 3D Slicer. Histogram equalization was performed to enhance image contrast by using the *AdaptiveHistogramEqualizationImageFilter* tool, also included in 3D Slicer, with the following parameter settings: α = 0.3, β = 0.3 and radius = 5.

The segmentation of the 146 fractured vertebrae was accomplished semiautomatically by one investigator (blinded) who has been trained by the senior radiologist. The vertebral body in the sagittal images was defined as the volume of interest (VOI), excluding the images without an apparent pedicle, and was segmented three-dimensionally using threshold- and erosion-based tools available in 3D Slicer. All segmentations were reviewed by the supervisor (blinded), and adjustments were made. Each segmentation was saved as an NRRD file, which is the native extension of the 3D Slicer. A dynamic representation of the segmentations can be seen in the electronic supplementary material.

### Three-Dimensional Extraction of Quantitative Features

**Fig. 1:**
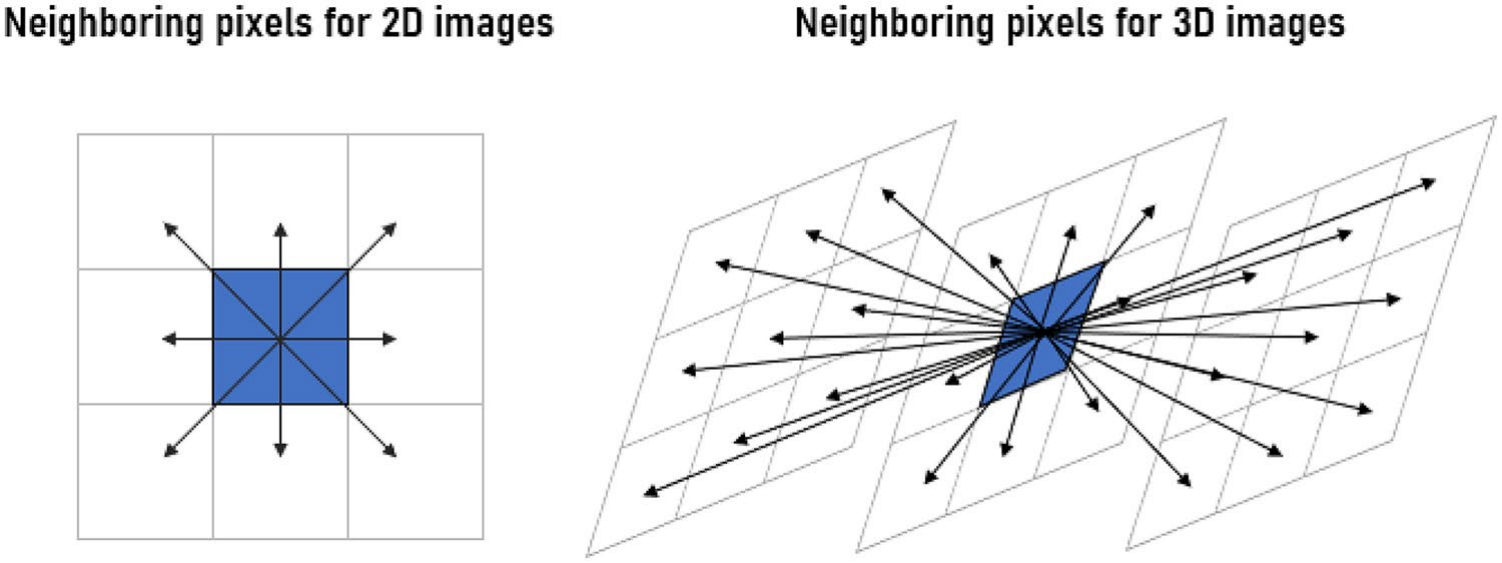
Neighborhood representation for a 2D and 3D feature extraction

Quantitative features were extracted from the VOIs by using PyRadiomics (https://pyradiomics.readthedocs.io/en/latest/*),* which is an open-source Python package. A Python script that imports the PyRadiomics package was coded to input the MRIs and segmentations, extract the radiomic features, normalize them to range from 0 to 1, and save them into a.csv file. Three-dimensionality was handled in this work in different ways for each feature category. Regarding the first-order features, they are calculated based on a histogram that represents the frequency distribution of the gray level intensities of the image. In this case, the three-dimensionality lies in the frequency distribution considering the gray level intensities of all slices of the volume of interest. Concerning texture attributes, they are traditionally calculated based on intensities variations between neighboring pixels. In the case of 2D feature extraction, this neighborhood is defined considering one single slice. In a 3D feature extraction, the neighborhood contains pixels from the other slices, and their relationships during the calculation are computed. Figure 1 shows the neighborhood representation for a 2D and 3D feature extraction considering the distance between pixels equal to 1.

Shape features were not extracted in this work, as the number of slices used for the three-dimensional characterization of each vertebral body was defined based on the presence or absence of pedicles. This intentional selection of slices could insert a bias during the shape analysis. The electronic supplementary material can show details of the 93 extracted features (19 first-order features and 74 texture features), which were calculated in accordance with the Image Biomarker Standardization Initiative (IBSI) [20].

### Model

Machine learning step was performed by using WEKA version 3.8 (https://www.cs.waikato.ac.nz/ml/weka/*)*. For classification, the *multilayer perceptron (MLP)* neural network with the *back-propagation* algorithm was chosen. Although deep neural networks have been state-of-the-art in the image classification task, we chose the *MLP* to get greater control over the features extracted from the images once our main objective was to find a radiomic signature that could bring more clarity to the radiologist's interpretation. As input data of the model, the.csv file containing the 93 features extracted from the segmented vertebral bodies was used so that each line corresponded to one fractured vertebral body and each column to a feature, the last one being designated for the label benign or malignant. The hyperparameters that WEKA allows to handle were set as follows: learning rate = 0.3, momentum = 0.2, batch size = 100, and epochs = 500, which are the default configuration. Other configurations were tested using the function *CVParameterSelection*, which is included in WEKA; however, they did not overcome the performance that used the default configuration. Concerning the hidden layers, they were set to correspond to the expression (number of features + number of classes) / 2. Therefore, during the feature selection steps, this hyperparameter was continuously changed according to the number of resulting features. Finally, the threshold used to compute the metrics was 0.5, which is the WEKA default.

### Experimental Design

**Fig. 2:**
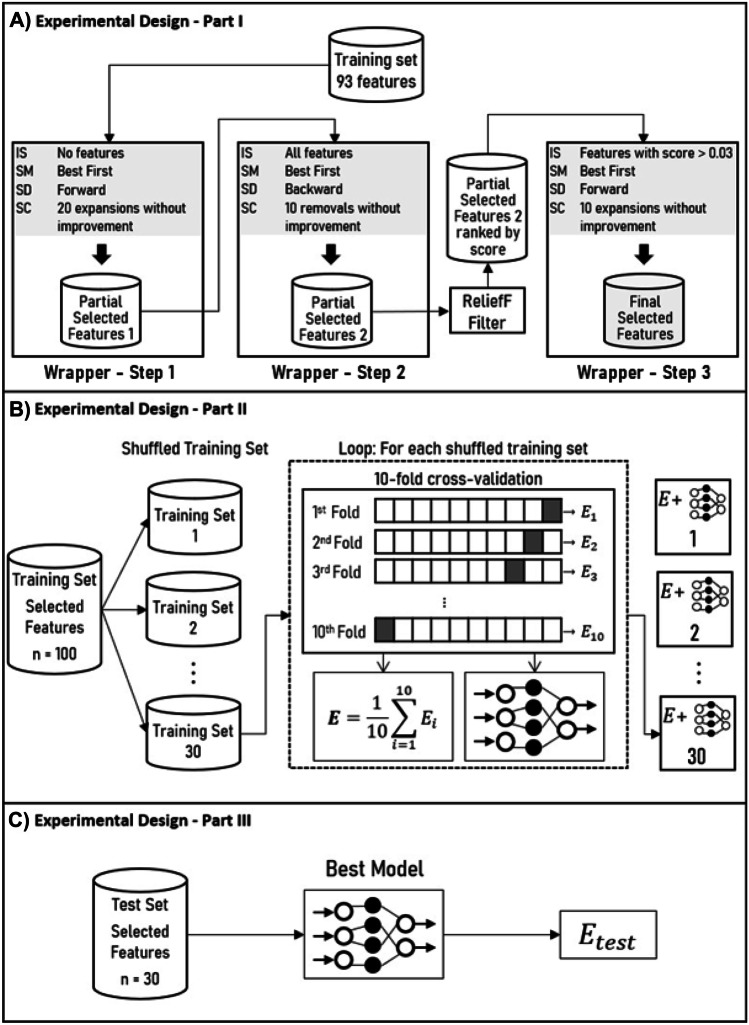
Experimental design step. **A** Feature selection procedure consisting of three steps. **B** Shows the internal validation test, which used 30 training sets with the previously selected features (obtained from original training set shuffle) and the tenfold cross-validation method to yield 30 neural network models. **C** Depicts the validation test that used both the model with the best evaluation in **(B)** and an independent test set to perform the final evaluation. E = evaluation. IS = initial state. SM = search method. SD = search direction. SC = stopping criterion

The experimental design consisted of three parts, as depicted in Fig. 2: i) feature selection, ii) internal validation test, and iii) independent validation test.

#### Feature Selection

To select the relevant features, the *wrapper* method was used. The *wrapper* consists of using a learning algorithm as a black box to evaluate the subset of features resulting from the search algorithm [21]. The parameters available in WEKA to configure the wrapper method are initial state, search method, search direction, stopping criterion, and evaluation metric. In the initial state, we must inform how the feature vector will be started. It can be empty, complete, or with just a few attributes. As a search method, WEKA makes available the *GreedyStepwise* or *BestFirst* options. Both algorithms perform a greedy search in the attribute space. However, in *GreedyStepwise*, the search is interrupted when an added (or removed) feature reduces the performance. In the case of *BestFirst*, a backtracking function is added to the algorithm allowing to compare the performance of each added/removed attribute with a defined number of past performances. Search direction can be set as forward, backward, or bi-directional. Forward is used when the initial state is empty, and the features are added to the subset of attributes to be evaluated. The backward option is used when the initial state is complete, and the features are deleted one by one during the process. With the bi-directional option, two simultaneous searches are carried out, one progressive and one regressive, with the aim of meeting in the middle of the path. The stopping criterion specifies how many attributes must be analyzed consecutively without improving the algorithm's performance. When that number is reached, the search stops. Finally, the evaluation metric is the measure that is used to compare the algorithm's performance for each subset of features.

In this work, we used the wrapper method in three steps. The *MLP* algorithm (previously specified) with tenfold cross-validation was chosen as the black box for all the steps. In addition, the *BestFirst* algorithm was selected as the search method, and the AUC ROC evaluation metric was picked to evaluate the performance of the features subset. The remaining parameters were set as follows:

- First Step: initial state = empty, search direction = forward, stopping criterion = 20 consecutive expansions without improvement, and evaluation metric = AUC ROC.
- Second Step: initial state = complete (all resulting features from the previous step), search direction = backward, stopping criterion = 10 consecutive removals without improvement, and evaluation metric = AUC ROC

To set the initial state of the last step, the attributes resulting from the second step were ranked using the ReliefF filter. Thus, those with a score > 0.03 were chosen to initialize the feature vector in the third step.

- Third Step: initial state = features with ReliefF score > 0.03, search direction = forward, stopping criterion = 10 consecutive expansions without improvement, and evaluation metric = AUC ROC

Figure 2A depicts this process.

#### Internal Validation Test

The training set containing 100 VCFs (54 benign and 46 malignant) with the features subset resulting from the feature selection step was used for internal validation. To assess the robustness of the radiomic features found in the feature selection process, the training set was shuffled 30 times. For each of these datasets, a new model was trained and validated using a tenfold cross-validation. Although the k-fold cross-validation technique is widely used to assess the generalization of a model, when it is performed only once on the training set, bias related to the split of the dataset may occur. This means that for a single execution, by chance, the data set could be split in a way that would generate the best classification, thus showing a resulting performance above the real averages. A previous random shuffle can minimize such bias. Overall performance was evaluated using the averages of accuracy, AUC ROC, sensitivity, and specificity. Figure 2B shows this process.

#### Independent Validation Test

The independent validation test aimed to evaluate the performance of the best model resulting from the internal validation test. For this, an independent test set with 30 VCFs (15 benign and 15 malignant), which had not been used at any time previously, was used. Data from this set were classified in a single run of the algorithm and the measures of accuracy, AUC ROC, sensitivity, and specificity were computed. Figure 2C depicts this process.

### Evaluation

The performance of the models was evaluated in terms of accuracy, area under the receiver operating characteristic curve (ROC AUC), sensitivity (corresponding to the true malignancy rate -TMR), and specificity (corresponding to the true benign rate—TBR). To evaluate the performance of the classifier over the 30 datasets in the internal validation test, the average with a 95% confidence interval for each of the above measures was used. To compare the accuracy, sensitivity, and specificity of the best model between internal and independent validation the hypothesis test for difference in two proportions was used.

## Results

### Patient Characteristics

**Fig. 3:**
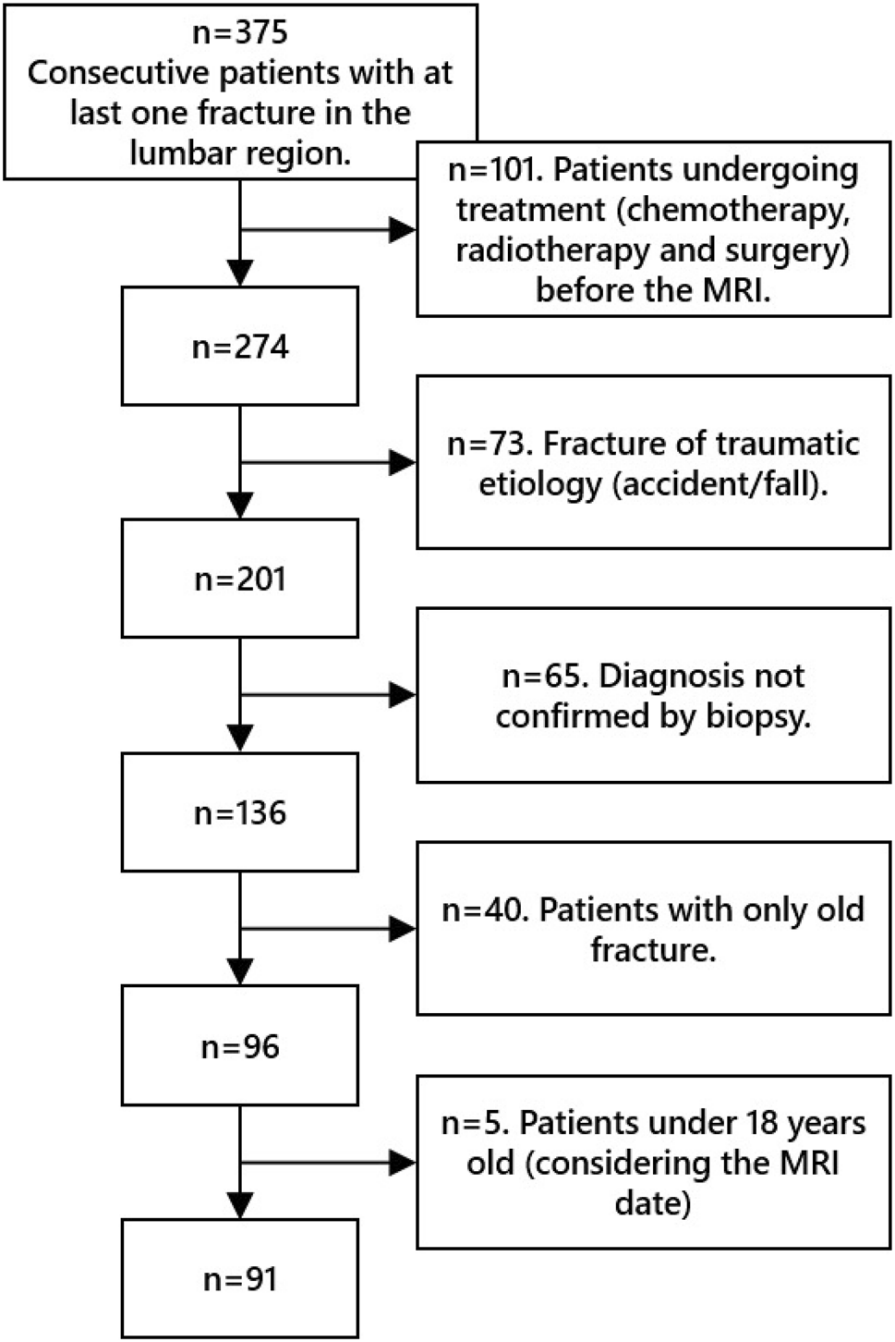
Diagram for inclusion/exclusion of the patients

A flowchart indicating the number of patients excluded for each exclusion criterion is shown in Fig. 3, and detailed demographic and clinical information is provided in Table 1. Of the 91 patients included in this study, 47 (52%) were diagnosed with fractures secondary to osteoporosis and 44 (48%) were diagnosed with fractures due to cancer. The most prevalent cancer type in the dataset was multiple myeloma (16.5%), followed by breast cancer (12%) and lung cancer (3.3%).

**Table 1:** Demographic and Clinical Characteristics of the Included Patients

Parameter	Training Group	Test Group	P-Value
Nº patients	61	30	
Age	63.2 ± 12.5	66.4 ± 9.9	0.19
Sex^a^			
Man	25/61 (41)	11/30 (37)	0.69
Woman	36/61 (59)	19/30 (63)	0.83
Nº patients per diagnosis^a^			
Osteoporosis (Benign Group)	32/61 (52)	15/30 (50)	0.83
Cancer (Malignant Group)	29/61 (48)	15/30 (50)	0.83
Multiple Myeloma	12/29 (41)	3/15 (20)	…
Breast Cancer	8/29 (28)	3/15 (20)	…
Lung Cancer	2/29 (7)	1/15 (7)	…
Squamous Cell Carcinoma	2/29 (7)	0/15 (0)	…
Prostate Cancer	1/29 (3)	1/15 (7)	…
Throat Cancer	0/29 (0)	2/15 (13)	…
Inflammatory Myofibroblastic Leukemia	1/29 (3)	0/15 (0)	…
Cholangiocarcinoma	1/29 (3)	0/15 (0)	…
Chronic Lymphocytic Leukemia	1/29 (3)	0/15 (0)	…
Paraganglioma	1/29 (3)	0/15 (0)	…
Thyroid Cancer	0/29 (0)	1/15 (7)	…
Testicular Cancer	0/29 (0)	1/15 (7)	…
Bladder Cancer	0/29 (0)	1/15 (7)	…
Kidney Cancer	0/29 (0)	1/15 (7)	…
Non-Hodgkin’s Lymphoma	0/29 (0)	1/15 (7)	…
Nº patients per nº fractured lumbar vertebrae^a^			
1 Fractured vertebra	39/61 (64)	22/30 (73)	0.35
2 Fractured vertebrae	12/61 (20)	4/30 (13)	0.43
3 Fractured vertebrae	6/61 (10)	1/30 (3)	0.20
4 Fractured vertebrae	1/61 (2)	2/30 (7)	0.30
5 Fractured vertebrae	3/61 (5)	1/30 (3)	0.71
Nº fractured vertebrae	100	46	
Nº fractured vertebrae used in the ML	100	30	
Nº fractured vertebrae per class^a^			
Benign fracture	54/100 (54)	15/30 (50)	0.70
Malignant fracture	46/100 (46)	15/30 (50)	0.70
Nº fractured vertebrae per type^a^			
L1	29/100 (29)	6/30 (20)	0.30
L2	19/100 (19)	6/30 (20)	0.90
L3	19/100 (19)	5/30 (17)	0.77
L4	19/100 (19)	9/30 (30)	0.23
L5	14/100 (14)	4/30 (13)	0.93

*ML* machine learning, *L* lumbar

^a^Data in parentheses are percentages

### Feature Selection

After the first run of the wrapper, 16 features were selected from the 93 initially extracted, as shown in Table 2. Of these 16 attributes, one was first-order-based, and 15 were texture-based. After the execution of the second step of the wrapper, the glrlm_ShortRunEmphasis, glszm_LowGrayLevelZoneEmphasis, and glszm_ZoneVariance features were removed. The remaining 13 attributes from Step 2 (Table 3) were then ranked using the ReliefF filter, and their scores were computed as shown in Table 4. Features that scored > 0.03 after running the ReliefF filter (which were ngtdm_Busyness, firstorder_10Percentile, ngtdm_Strength, and gldm_LowGrayLevelEmphasis) initialized the feature vector in the execution of the third and last wrapper running, whose result is shown in Table 5. These ten features selected at the end of the entire feature selection process are the variables we aimed to find. In this work, this set of features we are denominating as radiomic signature.

**Table 2:** Selected features after the first step of the wrapper method

firstorder_10Percentile
glcm_Id
glcm_Idm
glcm_InverseVariance
gldm_DependenceNonUniformityNormalized
gldm_LowGrayLevelEmphasis
gldm_SmallDependenceLowGrayLevelEmphasis
glrlm_LongRunLowGrayLevelEmphasis
glrlm_ShortRunEmphasis
glrlm_ShortRunLowGrayLevelEmphasis
glszm_GrayLevelNonUniformityNormalized
glszm_HighGrayLevelZoneEmphasis
glszm_LowGrayLevelZoneEmphasis
glszm_ZoneVariance
ngtdm_Busyness
ngtdm_Strength

**Table 3:** Selected features after the second step of the wrapper method

firstorder_10Percentile
glcm_Id
glcm_Idm
glcm_InverseVariance
gldm_DependenceNonUniformityNormalized
gldm_LowGrayLevelEmphasis
gldm_SmallDependenceLowGrayLevelEmphasis
glrlm_LongRunLowGrayLevelEmphasis
glrlm_ShortRunLowGrayLevelEmphasis
glszm_GrayLevelNonUniformityNormalized
glszm_HighGrayLevelZoneEmphasis
ngtdm_Busyness
ngtdm_Strength

**Table 4:** Ranking of features after executing the ReliefF filter

Average score	Feature
0.067 ± 0.007	ngtdm_Busyness
0.061 ± 0.008	firstorder_10Percentile
0.046 ± 0.004	ngtdm_Strength
0.031 ± 0.006	gldm_LowGrayLevelEmphasis
0.023 ± 0.003	glrlm_LongRunLowGrayLevelEmphasis
0.023 ± 0.007	glrlm_ShortRunLowGrayLevelEmphasis
0.02 ± 0.005	glszm_GrayLevelNonUniformityNormalized
0.014 ± 0.002	glcm_Idm
0.015 ± 0.005	gldm_SmallDependenceLowGrayLevelEmphasis
0.014 ± 0.002	glcm_Id
0.015 ± 0.004	glcm_InverseVariance
0.014 ± 0.004	glszm_HighGrayLevelZoneEmphasis
0.007 ± 0.003	gldm_DependenceNonUniformityNormalized

**Table 5:** Radiomic Signature

**Feature class**	**Feature names and descriptions**
First Order	**10th Percentile:** The 10th percentile of the sample
GLCM	**Inverse Difference:** It is a measure of the local homogeneity of an image. The higher the feature score is, the more homogeneous the image **Inverse Difference Moment:** Similar to the Inverse Difference, but with higher weights for elements that are close to the main diagonal **Inverse Variance:** Measures the heterogeneity of the image
GLDM	**Low Gray Level Emphasis:** Measures the distribution of low gray-level values, with a higher value indicating a greater concentration of low gray-level values in the image **Small Dependence Low Gray Level Emphasis:** Measures the joint distribution of small dependence with lower gray-level values
GLSZM	**Gray Level Nonuniformity Normalized:** Measures the variability of gray-level intensity values in the image, with a lower value indicating greater similarity in intensity values **High Gray Level Zone Emphasis:** Measures the distribution of the higher gray-level values, with a higher value indicating a greater proportion of higher gray-level values and size zones in the image
NGTDM	**Busyness:** It is a measure of the change from a pixel to its neighbor. A high value for busyness indicates a ‘busy’ image, with rapid changes in intensity between a pixel and its neighborhood **Strength:** It is a measure of the primitives in an image. Its value is high when the primitives are easily defined and visible, i.e., an image with a slow change in intensity but larger coarse differences in gray level intensities

*GLCM* gray level cooccurrence matrix, *GLDM* gray level dependence matrix, *GLSZM* gray level size zone matrix, *NGTDM* neighboring gray tone difference matrix

### Internal Validation Test

**Fig. 4:**
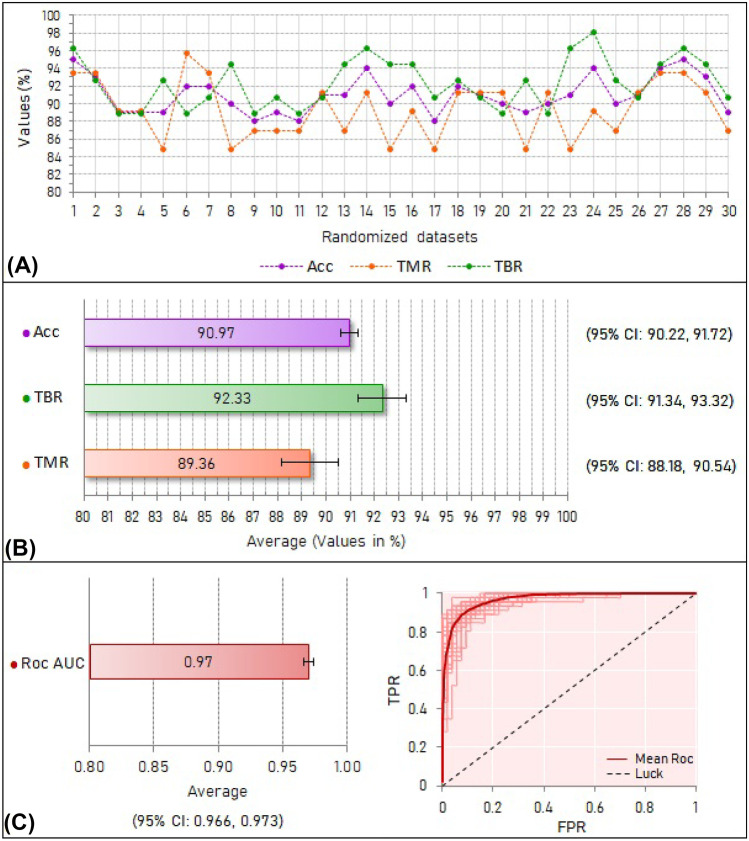
**A** Performance measurements for each randomized training dataset using tenfold cross-validation. **B** Average and 95% confidence interval of each performance measurement for all randomized datasets. Acc = accuracy. TBR = true benign rate (true negative rate). TMR = true malignancy rate (true positive rate). **C** Average with 95% confidence interval of the area under the receiver operating characteristic curve (ROC AUC) and receiver operating characteristic curve for all randomized datasets and their mean. TPR = true positive rate (true malignancy rate). FPR = false positive rate (false malignancy rate)

Figure 4 presents the results obtained in the internal validation test. Figure 4A plots the accuracy, true malignancy rate (sensitivity), and true benign rate (specificity) for each of the 30 models trained with tenfold cross-validation. The averages for accuracy, sensitivity, and specificity were as follows: 90.97% (95% CI: 90.22%, 91.72%), 89.36% (95% CI: 88.18%, 90.54%), and 92.33% (95% CI: 91.34%, 93.32%), respectively. These results are displayed in Fig. 4B. Figure 4C shows the average ROC AUC calculated for the 30 models, which was 0.97 (95% CI: 0.966, 0.974), and the plot of the ROC curves generated for each model.

**Fig. 5:**
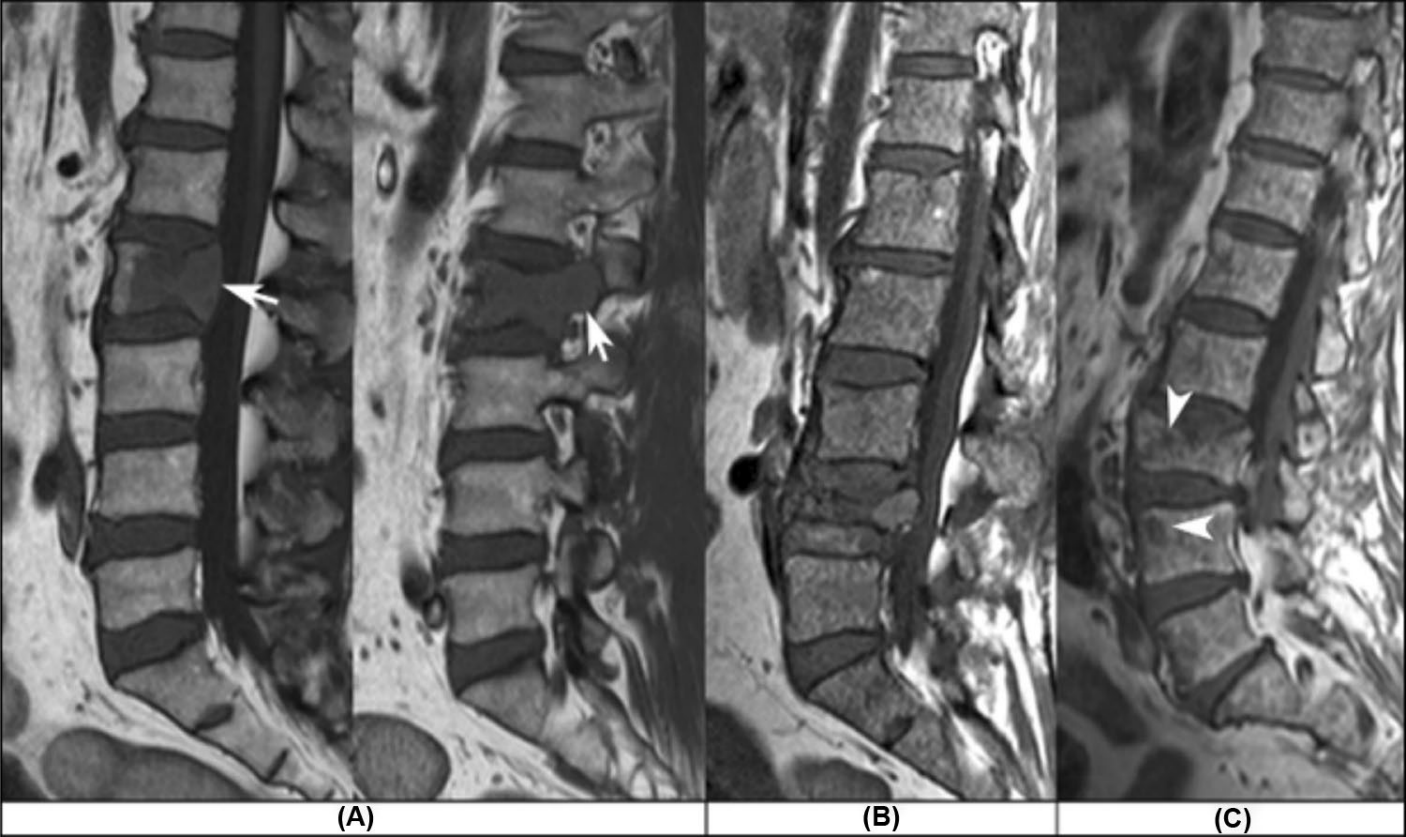
Illustration of the false-negative cases in the internal validation test. The three cases were of patients with multiple myeloma. In **A** (a 59-year-old man), the compressive vertebral fracture is associated with a focal nodular lesion, posterior wall convex bulging (left white arrow) and pedicle involvement (right white arrow). In **B** (a 52-year-old woman), and **C** (a 51-year-old woman), the bone marrow exhibits a salt-and-pepper infiltration pattern. The micronodules are better seen in **(C)** (white arrowheads). The L4 vertebral body fracture in **(B)** shows posterior wall retropulsion

The best model of the 30 yielded values for accuracy, ROC AUC, sensitivity, and specificity were as follows: 95% (95 of 100), 0.98, 93.5% (43 of 46), and 96.3% (52 of 54), respectively. The false negatives are depicted in Fig. 5.

### Independent Validation Test

**Fig. 6:**
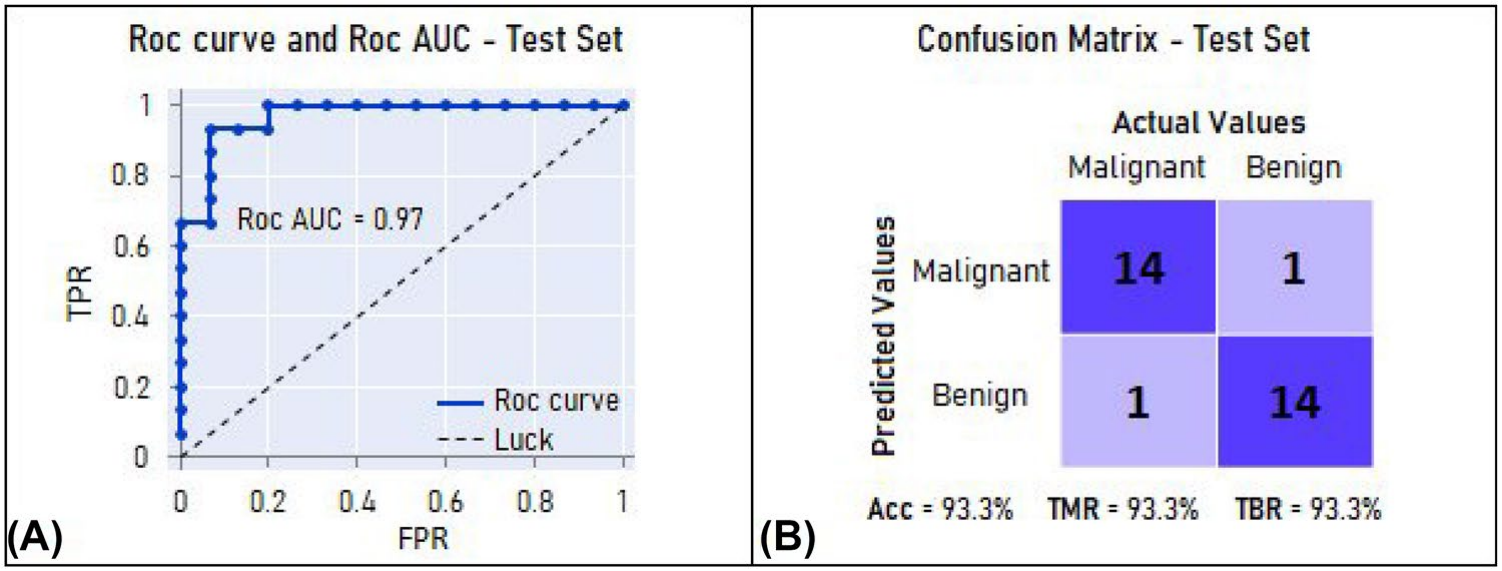
**A** ROC curve and ROC AUC of test set classification. **B** Confusion matrix of test set classification. ROC AUC = area under the receiver operating characteristic curve. TPR = true positive rate (true malignancy rate). FPR = false positive rate (false malignancy rate). Acc = accuracy. TMR = true malignancy rate. TBR = true benign rate

To perform this validation test, the independent test set was used to reevaluate the best model from the internal validation test. The accuracy, ROC AUC, true malignancy rate (sensitivity), and true benign rate (specificity) of the test classification were as follows: 93.3% (28/30), 0.97, 93.3% (14/15), and 93.3% (14/15), respectively. These results are presented in Fig. 6.

Table 6 shows the results of the hypothesis test to compare the accuracy, sensitivity, and specificity of the best model between internal and independent validation. The p-value for all metrics was > 0.05, meaning the model was not overfitting and could generalize learning.

**Table 6:** Internal versus Independent Validation

Metric	Internal Validation	Independent Validation	Estimate for difference	95% CI for difference	P-Value
Accuracy	0.950	0.933	0.017	(-0.082; 0.116)	0.741
Sensitivity	0.935	0.933	0.001	(-0.144; 0.146)	0.984
Specificity	0.963	0.933	0.030	(-0.106; 0.166)	0.669

## Discussion

In this study, the ability of the radiomics approach to differentiate benign from malignant VCFs using MRI was investigated. Our results showed high average values with narrow confidence intervals for the training set, indicating that the generated models were robust and the selected features were stable. The best proposed model had excellent performance for both the training set, using tenfold cross-validation, and the test set. Regarding performance based on tenfold cross-validation, the model resulted in just five instances of misclassification (two false positives and three false negatives). The false-negative cases were VCFs secondary to multiple myeloma. In two of these cases, the bone marrow exhibited a salt-and-pepper infiltration pattern, and we presume that the neural network would need more cases in the MRI database to reach appropriate training to recognize this specific pattern.

Concerning the diagnostic performance achieved using the independent test set, the model misclassified just one instance of each class. It is worth noting that in classification models applied to health care, a false negative-type error is more worrying than a false positive, because the former would delay or prevent treatment. False negatives become even more critical when dealing with diseases such as cancer, where early diagnosis is essential for improving patient survival [22].

Regarding radiomic signature, of the ten characteristics selected in the feature selection step, nine were texture-based, and one was intensity-based. This shows that the patterns found by the neural network are more related to the spatial relationship of the voxels than to their intensity values. Such a phenomenon was expected since, in contrast to CT images, which allow for a correlation between the signal intensity and the X-ray attenuation coefficient of the tissue, in MR images, this is not possible due to the nature of their acquisition. In this case, the signal intensities arise from the interaction between tissue properties, such as relaxation time, and acquisition parameters. Two features that stood out in this study were busyness (or fineness) and strength. A busy texture occurs when there are fast intensity changes between a pixel/voxel and its neighbors. Strength refers to how easily a primitive can be clearly definable and visible [23]. The results showed that malignant VCFs tended to have high busyness and low strength, while benign VCFs tended to show the opposite features.

To the best of our knowledge, our work yielded better results than previous studies that used computer-aided techniques in MRI to differentiate benign from malignant VCFs. Frighetto-Pereira et al. [14] achieved a ROC AUC of 0.91 and an accuracy of 86.41%. Azevedo-Marques et al. [15] used k-NN and tenfold cross-validation and obtained an average accuracy of 82.9% and an average ROC AUC of 0.81. Frighetto et al. [16] achieved an accuracy of 85.3% and a ROC AUC of 0.92. Casti et al. [17] obtained an accuracy of 92% using the QDA, SVM, and k-NN classifiers and a ROC AUC of 0.95 (with QDA). Finally, Arpitha and Rangarajan [18] proposed a segmentation and classification method based on shape and texture to differentiate benign from malignant VCFs. The authors did not use accuracy and AUC for evaluation, but it was possible to estimate accuracy from the recall value and the number of vertebral bodies used, yielding a value of approximately 93.1%.

In a recent study, Chee et al. [24] evaluated the prediction of malignancy in vertebral fractures using a combined radiomics-clinical model on CT. Although such a model also performed a 3D evaluation, our results outperform theirs considering both the radiomics model alone and the combined model. In addition, as the authors themselves mention, MRI is the modality of choice for the differential diagnosis of vertebral compression fractures since it is more sensitive for detecting neoplastic bone marrow infiltration. Another substantial advantage of MRI is that it does not use ionizing radiation.

There are limitations to our study. We used only T1-weighted sagittal images. The vertebral bodies of the thoracic and cervical regions were not evaluated. Although evaluation with an independent test set was performed, it consisted of patients from the same institution as the training set. The segmentation is a limitation of our study as well. We used a semiautomatic method; however, to implement the methods proposed in clinical practice, it would be desirable to have a fully automatic segmentation method. Some studies have focused their efforts on developing these tools [18, 25–27]. Our future efforts in this regard will be directed to the use of U-Net networks [28] to segment VCFs. Although the ground truth has been defined based on tests of high sensitivity and specificity, which are biopsies (for malignant fractures) and DEXA scan with a follow-up of at least 2 years (for benign fractures), only one person checking the reports was a limitation.

In conclusion, the neural network-based model proposed in this study to differentiate benign from malignant vertebral compression fractures achieved excellent diagnostic performance, overcoming the limitations of previous studies. The insertion of this model in the clinical routine would have potential benefits for patients, radiologists, and health institutions. For patients, the early diagnosis, and the decrease of invasive exams, such as biopsies. For radiologists, the work optimization, and the increase in visibility of their role in personalized medicine. For health institutions, the cost reduction due to the decrease in invasive exams and the recognition due to the technological differential.

## Supplementary Information

Below is the link to the electronic supplementary material.

Supplementary file1 (DOCX 2400 KB)

## Data Availability

The data present in this submission have not yet been published, but we intend to publish them soon. This article will be cited in the database publication.

## Abbreviations

AUC: Area under the curve
CAD: Computer-aided diagnosis
CI: Confidence interval
CT: Computed tomography
DICOM: Digital imaging and communications in medicine
FOV: Field of view
GLCM: Gray level cooccurrence matrix
GLDM: Gray level dependency matrix
GLSZM: Gray level size zone matrix
HIS: Hospital information system
K-NN: K-nearest neighbors
LDA: Linear discriminant analysis
MRI: Magnetic resonance imaging
MLP: Multilayer Perceptron
NGTDM: Neighboring gray tone difference matrix
PACS: Picture archiving and communication system
QDA: Quadratic discriminant analysis
RBF: Radial basis function
RIS: Radiology information system
ROC: Receiver operating characteristic
SVM: Support vector machine
T1WI: T1-weighted image
T2WI: T2-weighted image
TE: Time of echo
TR: Time of repetition
VCF: Vertebral compression fracture
VOI: Volume of interest

## References

[1] Alexandru D, So W (2012). Evaluation and Management of Vertebral Compression Fractures. Perm J.

[2] Cuénod CA, Laredo JD, Chevret S (1996). Acute vertebral collapse due to osteoporosis or malignancy: appearance on unenhanced and gadolinium-enhanced MR images. Radiology.

[3] Lecouvet FE, Vande Berg BC, Maldague BE (1997). Vertebral compression fractures in multiple myeloma. Part I. Distribution and appearance at MR imaging. Radiology.

[4] Suh CH, Yun SJ, Jin W (2018). ADC as a useful diagnostic tool for differentiating benign and malignant vertebral bone marrow lesions and compression fractures: a systematic review and meta-analysis. Eur Radiol.

[5] Lecouvet FE (2016). Whole-Body MR Imaging: Musculoskeletal Applications. Radiology.

[6] Porter BA, Shields AF, Olson DO (1986). Magnetic resonance imaging of bone marrow disorders. Radiol Clin North Am.

[7] Mauch JT, Carr CM, Cloft H, Diehn FE (2018). Review of the Imaging Features of Benign Osteoporotic and Malignant Vertebral Compression Fractures. American Journal of Neuroradiology.

[8] Thawait SK, Marcus MA, Morrison WB, et al (2012) Research synthesis: what is the diagnostic performance of magnetic resonance imaging to discriminate benign from malignant vertebral compression fractures? Systematic review and meta-analysis. Spine (Phila Pa 1976) 37:E736–44. 10.1097/BRS.0b013e3182458cac 10.1097/BRS.0b013e3182458cac 22210011

[9] Tehranzadeh J, Tao C (2004). Advances in MR imaging of vertebral collapse. Seminars in Ultrasound, CT and MRI.

[10] Arana E, Kovacs FM, Royuela A (2020). Metastatic Versus Osteoporotic Vertebral Fractures on MRI: A Blinded, Multicenter, and Multispecialty Observer Agreement Evaluation. Journal of the National Comprehensive Cancer Network.

[11] Aerts HJWL, Velazquez ER, Leijenaar RTH (2014). Decoding tumour phenotype by noninvasive imaging using a quantitative radiomics approach. Nat Commun.

[12] Napel S, Mu W, Jardim-Perassi BV (2018). Quantitative imaging of cancer in the postgenomic era: Radio(geno)mics, deep learning, and habitats. Cancer.

[13] Gillies RJ, Kinahan PE, Hricak H (2016). Radiomics: Images Are More than Pictures, They Are Data. Radiology.

[14] Frighetto-Pereira L, Menezes-Reis R, Metzner GA, et al (2015) Semiautomatic classification of benign versus malignant vertebral compression fractures using texture and gray-level features in magnetic resonance images. In: 2015 IEEE 28th International Symposium on Computer-Based Medical Systems. IEEE, pp 88–92

[15] Azevedo-Marques PM, Spagnoli HF, Frighetto-Pereira L, et al (2015) Classification of vertebral compression fractures in magnetic resonance images using spectral and fractal analysis. In: 2015 37th Annual International Conference of the IEEE Engineering in Medicine and Biology Society (EMBC). IEEE, pp 723–726 10.1109/EMBC.2015.7318464 26736364

[16] Frighetto-Pereira L, Rangayyan RM, Metzner GA (2016). Shape, texture and statistical features for classification of benign and malignant vertebral compression fractures in magnetic resonance images. Comput Biol Med.

[17] Casti P, Mencattini A, Nogueira-Barbosa MH (2017). Cooperative strategy for a dynamic ensemble of classification models in clinical applications: the case of MRI vertebral compression fractures. Int J Comput Assist Radiol Surg.

[18] Arpitha Adela, Rangarajan L (2020). Computational techniques to segment and classify lumbar compression fractures. Radiol Med.

[19] Ortiz-Ramon R, Larroza A, Arana E, Moratal D (2017) A radiomics evaluation of 2D and 3D MRI texture features to classify brain metastases from lung cancer and melanoma. In: 2017 39th Annual International Conference of the IEEE Engineering in Medicine and Biology Society (EMBC). IEEE, pp 493–496 10.1109/EMBC.2017.8036869 29059917

[20] Zwanenburg A, Vallières M, Abdalah MA (2020). The Image Biomarker Standardization Initiative: Standardized Quantitative Radiomics for High-Throughput Image-based Phenotyping. Radiology.

[21] Kohavi R, John GH (1997). Wrappers for feature subset selection. Artif Intell.

[22] Paci E, Ponti A, Zappa M (2005). Early diagnosis, not differential treatment, explains better survival in service screening. Eur J Cancer.

[23] Amadasun M, King R (1989). Textural features corresponding to textural properties. IEEE Trans Syst Man Cybern.

[24] Chee CG, Yoon MA, Kim KW (2021). Combined radiomics-clinical model to predict malignancy of vertebral compression fractures on CT. Eur Radiol.

[25] Ramos JS, Cazzolato MT, Nogueira-Barbosa MH, Traina AJM (2020) FINE. In: Proceedings of the 35th Annual ACM Symposium on Applied Computing. ACM, New York, NY, USA, pp 198–201

[26] Ramos JS, Watanabe CY V., Nogueira-Barbosa MH, Traina AJM (2019) BGrowth. In: Proceedings of the 34th ACM/SIGAPP Symposium on Applied Computing. ACM, New York, NY, USA, pp 220–227

[27] Rak M, Steffen J, Meyer A (2019). Combining convolutional neural networks and star convex cuts for fast whole spine vertebra segmentation in MRI. Comput Methods Programs Biomed.

[28] Ronneberger O, Fischer P, Brox T (2015) U-Net: Convolutional networks for biomedical image segmentation. In: Proceedings of the Medical Image Computing and Computer-Assisted Intervention–MICCAI 2015. Munich, Germany, pp. 234–241. 10.1007/978-3-319-24574-4_28

