# Supplementary material for: A 3D Radiomics-Based Artificial Neural Network Model for Benign Versus Malignant Vertebral Compression Fracture Classification in MRI
Source: J Digit Imaging. 2023 May 30;36(4):1565–77. doi: 10.1007/s10278-023-00847-4 (PMC10406770; doi:10.1007/s10278-023-00847-4)
Supplement: Supplementary file 1 — Supplementary file1 (DOCX 2400 KB) [file 10278_2023_847_MOESM1_ESM.docx]

**Electronic Supplementary Material**

[MR parameters of T1-weighted images 1](#_Toc133829428)

[Dynamic representation of the segmentation 3](#_Toc133829429)

[Extracted Features 5](#_Toc133829430)

# MR parameters of T1-weighted images

| Exam | TE (ms) | TR (ms) | FOVx (mm) | FOVy (mm) | Number of Averages | Pixel Bandwidth (hz/pixel) | Slice Thickness (mm) | Spacing between Slices (mm) | Magnetic Field Strength (T) | Rows | Columns | Pixel Size (mm^2^) | Resolution (pixel/mm) | Number  of Slices |
| --- | --- | --- | --- | --- | --- | --- | --- | --- | --- | --- | --- | --- | --- | --- |
| P1 | 10 | 400 | 334.8 | 334.8 | 4 | 217 | 4 | 4 | 1.5 | 640 | 640 | 0.5233 | 1.9111 | 14 |
| P2 | 10 | 400 | 338.8 | 338.8 | 4 | 221 | 4 | 4 | 1.5 | 640 | 640 | 0.5294 | 1.8889 | 14 |
| P3 | 10.2 | 478.3 | 350 | 350 | 3 | 225 | 4 | 4.4 | 1.5 | 896 | 896 | 0.3906 | 2.56 | 12 |
| P4 | 10 | 400 | 338.8 | 338.8 | 4 | 221 | 4 | 4 | 1.5 | 640 | 640 | 0.5294 | 1.8889 | 14 |
| P5 | 10.2 | 478.3 | 330 | 330 | 3 | 214 | 4 | 4.4 | 1.5 | 864 | 864 | 0.3819 | 2.6182 | 12 |
| P6 | 10.2 | 478.3 | 330 | 330 | 3 | 214 | 4 | 4.4 | 1.5 | 864 | 864 | 0.3819 | 2.6182 | 12 |
| P7 | 10.2 | 478.3 | 330 | 330 | 3 | 214 | 4 | 4.4 | 1.5 | 864 | 864 | 0.3819 | 2.6182 | 12 |
| P8 | 8 | 675.3 | 202.5 | 202.5 | 2 | 254 | 4 | 4 | 3 | 432 | 432 | 0.4688 | 2.1333 | 12 |
| P9 | 10 | 400 | 338.8 | 338.8 | 4 | 210 | 4 | 4 | 1.5 | 640 | 640 | 0.5294 | 1.8889 | 14 |
| P10 | 10.2 | 568.4 | 183.6 | 183.6 | 2 | 226 | 3 | 3 | 1.5 | 480 | 480 | 0.3827 | 2.6133 | 12 |
| P11 | 10.2 | 478.3 | 330 | 630.2 | 3 | 214 | 4 | 4.4 | 1.5 | 1650 | 864 | 0.3819 | 2.6182 | 12 |
| P12 | 10.2 | 478.3 | 330 | 330 | 3 | 207 | 4 | 4.4 | 1.5 | 864 | 864 | 0.3819 | 2.6182 | 12 |
| P13 | 10 | 400 | 334.8 | 334.8 | 3 | 206 | 4 | 4 | 1.5 | 640 | 640 | 0.5233 | 1.9111 | 14 |
| P14 | 10 | 400 | 338.8 | 338.8 | 4 | 221 | 4 | 4 | 1.5 | 640 | 640 | 0.5294 | 1.8889 | 14 |
| P15 | 10.2 | 478.3 | 320 | 320 | 3 | 225 | 4 | 4.4 | 1.5 | 864 | 864 | 0.3704 | 2.7 | 12 |
| P16 | 10.2 | 478.3 | 300 | 300 | 3 | 225 | 4 | 4.4 | 1.5 | 768 | 768 | 0.3906 | 2.56 | 12 |
| P17 | 7 | 441.5 | 340 | 340 | 3 | 374 | 3 | 3.3 | 3 | 704 | 704 | 0.483 | 2.0706 | 14 |
| P18 | 10.2 | 478.3 | 330 | 330 | 3 | 214 | 4 | 4.4 | 1.5 | 864 | 864 | 0.3819 | 2.6182 | 12 |
| P19 | 10.2 | 478.3 | 290 | 290 | 3 | 224 | 4 | 4.4 | 1.5 | 768 | 768 | 0.3776 | 2.6483 | 12 |
| P20 | 8 | 675.3 | 316.4 | 316.4 | 2 | 258 | 4 | 4 | 3 | 640 | 640 | 0.4945 | 2.0222 | 12 |
| P21 | 10.2 | 567.5 | 219.6 | 601.7 | 3 | 223 | 4 | 4 | 1.5 | 1578 | 576 | 0.3814 | 2.6222 | 12 |
| P22 | 7 | 441.5 | 340 | 340 | 3 | 408 | 3 | 3.3 | 3 | 704 | 704 | 0.483 | 2.0706 | 14 |
| P23 | 8 | 675.3 | 303.1 | 303.1 | 2 | 258 | 4 | 4 | 3 | 640 | 640 | 0.4737 | 2.1111 | 12 |
| P24 | 10.2 | 597.8 | 329.6 | 329.6 | 3 | 224 | 3.6 | 3.6 | 1.5 | 864 | 864 | 0.3816 | 2.6207 | 14 |
| P25 | 10.2 | 478.3 | 330 | 330 | 3 | 214 | 4 | 4.4 | 1.5 | 864 | 864 | 0.3819 | 2.6182 | 12 |
| P26 | 10.2 | 478.3 | 330 | 330 | 3 | 214 | 4 | 4.4 | 1.5 | 864 | 864 | 0.3819 | 2.6182 | 12 |
| P27 | 10 | 400 | 338.8 | 338.8 | 4 | 221 | 4 | 4 | 1.5 | 640 | 640 | 0.5294 | 1.8889 | 14 |
| P28 | 10.2 | 478.3 | 330 | 330 | 3 | 225 | 4 | 4.4 | 1.5 | 864 | 864 | 0.3819 | 2.6182 | 12 |
| P29 | 8 | 414.1 | 250 | 250 | 3 | 364 | 4 | 4.4 | 3 | 512 | 512 | 0.4883 | 2.048 | 12 |
| P30 | 10.2 | 478.3 | 353.5 | 353.5 | 3 | 222 | 4 | 4.4 | 1.5 | 960 | 960 | 0.3683 | 2.7152 | 12 |
| P31 | 10 | 400 | 338.8 | 338.8 | 4 | 221 | 4 | 4 | 1.5 | 640 | 640 | 0.5294 | 1.8889 | 14 |
| P32 | 10 | 400 | 338.8 | 338.8 | 4 | 221 | 4 | 4 | 1.5 | 640 | 640 | 0.5294 | 1.8889 | 14 |
| P33 | 10.2 | 478.3 | 280 | 280 | 3 | 213 | 4 | 4.4 | 1.5 | 720 | 720 | 0.3889 | 2.5714 | 12 |
| P34 | 10 | 400 | 334.8 | 334.8 | 3 | 217 | 4 | 4 | 1.5 | 640 | 640 | 0.5233 | 1.9111 | 14 |
| P35 | 10.2 | 478.3 | 330 | 330 | 3 | 214 | 4 | 4.4 | 1.5 | 864 | 864 | 0.3819 | 2.6182 | 12 |
| P36 | 10.2 | 478.3 | 352 | 352 | 3 | 214 | 4 | 4.4 | 1.5 | 960 | 960 | 0.3667 | 2.7273 | 12 |
| P37 | 10.2 | 478.3 | 352 | 352 | 3 | 214 | 4 | 4.4 | 1.5 | 960 | 960 | 0.3667 | 2.7273 | 12 |
| P38 | 10.2 | 478.3 | 330 | 330 | 3 | 214 | 4 | 4.4 | 1.5 | 864 | 864 | 0.3819 | 2.6182 | 12 |
| P39 | 10.2 | 478.3 | 330 | 330 | 3 | 214 | 4 | 4.4 | 1.5 | 864 | 864 | 0.3819 | 2.6182 | 12 |
| P40 | 10 | 400 | 339.7 | 339.7 | 3 | 203 | 4 | 4 | 1.5 | 672 | 672 | 0.5056 | 1.9778 | 14 |
| P41 | 10.2 | 478.3 | 330 | 330 | 3 | 214 | 4 | 4.4 | 1.5 | 864 | 864 | 0.3819 | 2.6182 | 12 |
| P42 | 10.2 | 478.3 | 300 | 300 | 3 | 225 | 4 | 4.4 | 1.5 | 768 | 768 | 0.3906 | 2.56 | 12 |
| P43 | 10.2 | 478.3 | 330 | 330 | 3 | 214 | 4 | 4.4 | 1.5 | 864 | 864 | 0.3819 | 2.6182 | 12 |
| P44 | 8 | 675.3 | 293.8 | 293.8 | 2 | 240 | 4 | 4 | 3 | 640 | 640 | 0.4592 | 2.1778 | 12 |
| P45 | 10.2 | 478.3 | 340 | 340 | 3 | 213 | 4 | 4.4 | 1.5 | 880 | 880 | 0.3864 | 2.5882 | 12 |
| P46 | 10.2 | 478.3 | 320 | 320 | 3 | 225 | 4 | 4.4 | 1.5 | 864 | 864 | 0.3704 | 2.7 | 12 |
| P47 | 10.2 | 478.3 | 350 | 350 | 3 | 210 | 4 | 4.4 | 1.5 | 896 | 896 | 0.3906 | 2.56 | 12 |
| P48 | 10.2 | 478.3 | 400 | 400 | 3 | 214 | 4 | 4.4 | 1.5 | 1024 | 1024 | 0.3906 | 2.56 | 12 |
| P49 | 10 | 584.5 | 338.8 | 338.8 | 4 | 236 | 4 | 4 | 1.5 | 640 | 640 | 0.5294 | 1.8889 | 12 |
| P50 | 10.2 | 478.3 | 330 | 330 | 3 | 214 | 4 | 4 | 1.5 | 864 | 864 | 0.3819 | 2.6182 | 12 |
| P51 | 10.2 | 478.3 | 330 | 330 | 3 | 214 | 4 | 4.4 | 1.5 | 864 | 864 | 0.3819 | 2.6182 | 12 |
| P52 | 10.2 | 478.3 | 300 | 300 | 4 | 221 | 4 | 4.4 | 1.5 | 768 | 768 | 0.3906 | 2.56 | 12 |
| P53 | 10.2 | 478.3 | 330.6 | 330.6 | 4 | 218 | 4 | 4.4 | 1.5 | 864 | 864 | 0.3827 | 2.6133 | 12 |
| P54 | 10 | 580.9 | 338.8 | 338.8 | 4 | 221 | 4 | 4.4 | 1.5 | 640 | 640 | 0.5294 | 1.8889 | 12 |
| P55 | 10 | 580.9 | 338.8 | 338.8 | 4 | 221 | 4 | 4.4 | 1.5 | 640 | 640 | 0.5294 | 1.8889 | 12 |
| P56 | 10 | 582.6 | 338.8 | 338.8 | 4 | 210 | 4 | 4.4 | 1.5 | 640 | 640 | 0.5294 | 1.8889 | 12 |
| P57 | 10 | 427.1 | 334.8 | 334.8 | 3 | 206 | 4 | 4.4 | 1.5 | 640 | 640 | 0.5233 | 1.9111 | 15 |
| P58 | 10 | 400 | 418.6 | 418.6 | 4 | 221 | 5 | 5.5 | 1.5 | 800 | 800 | 0.5233 | 1.9111 | 14 |
| P59 | 10 | 580.9 | 338.8 | 338.8 | 4 | 221 | 4 | 4.4 | 1.5 | 640 | 640 | 0.5294 | 1.8889 | 12 |
| P60 | 10.2 | 478.3 | 320 | 320 | 4 | 221 | 3 | 3 | 1.5 | 864 | 864 | 0.3704 | 2.7 | 12 |
| P61 | 10.2 | 478.3 | 330 | 330 | 3 | 214 | 4 | 4.4 | 1.5 | 864 | 864 | 0.3819 | 2.6182 | 12 |
| P62 | 11 | 570 | 280 | 280 | 3 | 160 | 4 | 5.2 | 1.5 | 384 | 384 | 0.7292 | 1.3714 | 12 |
| P63 | 14 | 425 | 330 | 330 | 4 | 287 | 3.6 | 4.6 | 1.5 | 768 | 768 | 0.4297 | 2.3273 | 12 |
| P64 | 12 | 432.5 | 338.8 | 338.8 | 4 | 262 | 4 | 4 | 1.5 | 640 | 640 | 0.5294 | 1.8889 | 14 |
| P65 | 10 | 400 | 338.8 | 338.8 | 4 | 221 | 4 | 4 | 1.5 | 640 | 640 | 0.5294 | 1.8889 | 14 |
| P66 | 12 | 431.7 | 338.8 | 338.8 | 6 | 270 | 4 | 4 | 1.5 | 640 | 640 | 0.5294 | 1.8889 | 14 |
| P67 | 10 | 400 | 360 | 360 | 4 | 214 | 4 | 4 | 1.5 | 704 | 704 | 0.5114 | 1.9556 | 14 |
| P68 | 12 | 430.1 | 334.8 | 334.8 | 4 | 278 | 4 | 4 | 1.5 | 640 | 640 | 0.5233 | 1.9111 | 14 |
| P69 | 10.2 | 568.1 | 228.5 | 625 | 3 | 220 | 3.5 | 3.5 | 1.5 | 1750 | 640 | 0.3571 | 2.8 | 12 |
| P70 | 7.338 | 441.5 | 316.4 | 316.4 | 2 | 387 | 4 | 4.4 | 3 | 640 | 640 | 0.4945 | 2.0222 | 14 |
| P71 | 14 | 425 | 330 | 330 | 3 | 287 | 3.6 | 4.6 | 1.5 | 768 | 768 | 0.4297 | 2.3273 | 12 |
| P72 | 14 | 425 | 330 | 330 | 3 | 287 | 3.6 | 4.6 | 1.5 | 768 | 768 | 0.4297 | 2.3273 | 12 |
| P73 | 14 | 573.3 | 330 | 330 | 4 | 662 | 4 | 5 | 1.5 | 768 | 768 | 0.4297 | 2.3273 | 12 |
| P74 | 14 | 425 | 300 | 300 | 4 | 287 | 3.6 | 4.6 | 1.5 | 704 | 704 | 0.4261 | 2.3467 | 12 |
| P75 | 10 | 606.6 | 317.4 | 317.4 | 3 | 178 | 4 | 4.4 | 3 | 640 | 640 | 0.496 | 2.016 | 12 |
| P76 | 12 | 431.7 | 338.8 | 338.8 | 6 | 270 | 4 | 4 | 1.5 | 640 | 640 | 0.5294 | 1.8889 | 14 |
| P77 | 7 | 441.5 | 340 | 340 | 3 | 408 | 3 | 3.3 | 3 | 704 | 704 | 0.483 | 2.0706 | 14 |
| P78 | 8 | 675.3 | 271.7 | 271.7 | 2 | 237 | 4.5 | 4.5 | 3 | 320 | 320 | 0.8491 | 1.1778 | 12 |
| P79 | 12 | 430.1 | 339.7 | 339.7 | 6 | 278 | 4 | 4 | 1.5 | 672 | 672 | 0.5056 | 1.9778 | 14 |
| P80 | 10.2 | 568.3 | 228.5 | 228.5 | 3 | 219 | 3.5 | 4.5 | 1.5 | 640 | 640 | 0.3571 | 2.8 | 12 |
| P81 | 12 | 431.8 | 334.8 | 334.8 | 6 | 265 | 4 | 5 | 1.5 | 640 | 640 | 0.5233 | 1.9111 | 14 |
| P82 | 7 | 441.5 | 340 | 340 | 3 | 408 | 3.5 | 3.8 | 3 | 704 | 704 | 0.483 | 2.0706 | 14 |
| P83 | 7 | 441.5 | 340 | 340 | 3 | 408 | 3 | 3.3 | 3 | 704 | 704 | 0.483 | 2.0706 | 14 |
| P84 | 12 | 430.0 | 338.8 | 338.8 | 6 | 284 | 4 | 4 | 1.5 | 640 | 640 | 0.5294 | 1.8889 | 14 |
| P85 | 12 | 430.9 | 300 | 300 | 8 | 277 | 4 | 4 | 1.5 | 576 | 576 | 0.5208 | 1.92 | 14 |
| P86 | 14 | 425 | 300 | 300 | 3 | 287 | 3.6 | 4.6 | 1.5 | 704 | 704 | 0.4261 | 2.3467 | 12 |
| P87 | 14 | 425 | 330 | 330 | 4 | 287 | 3.6 | 4.6 | 1.5 | 768 | 768 | 0.4297 | 2.3273 | 12 |
| P88 | 14 | 573.3 | 380 | 380 | 3 | 767 | 3.6 | 4.6 | 1.5 | 864 | 864 | 0.4398 | 2.2737 | 12 |
| P89 | 7 | 441.5 | 340 | 340 | 3 | 408 | 3 | 3.3 | 3 | 704 | 704 | 0.483 | 2.0706 | 15 |
| P90 | 12 | 431.8 | 334.8 | 334.8 | 4 | 265 | 4 | 4 | 1.5 | 640 | 640 | 0.5233 | 1.9111 | 14 |
| P91 | 14 | 424.9 | 350 | 350 | 3 | 287 | 3.6 | 4.6 | 1.5 | 800 | 800 | 0.4375 | 2.2857 | 12 |

# Dynamic representation of the segmentation

6
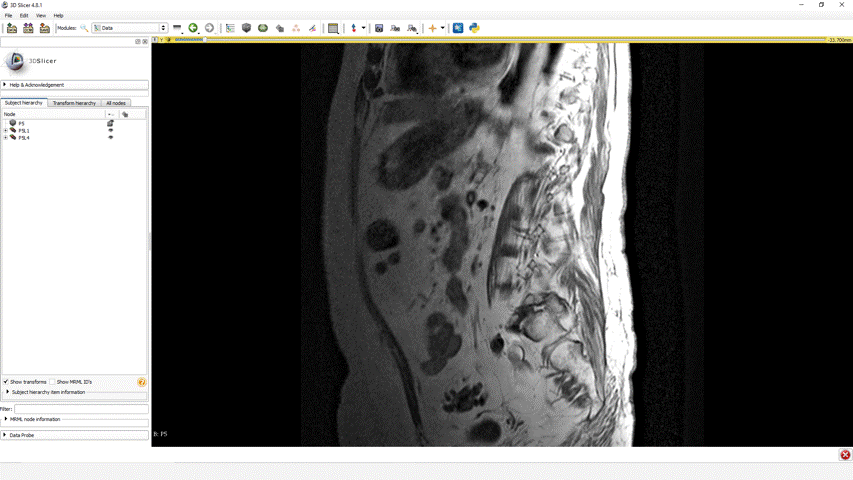


Figure 1 - Segmentation performed only on slices that did not have an apparent pedicle.


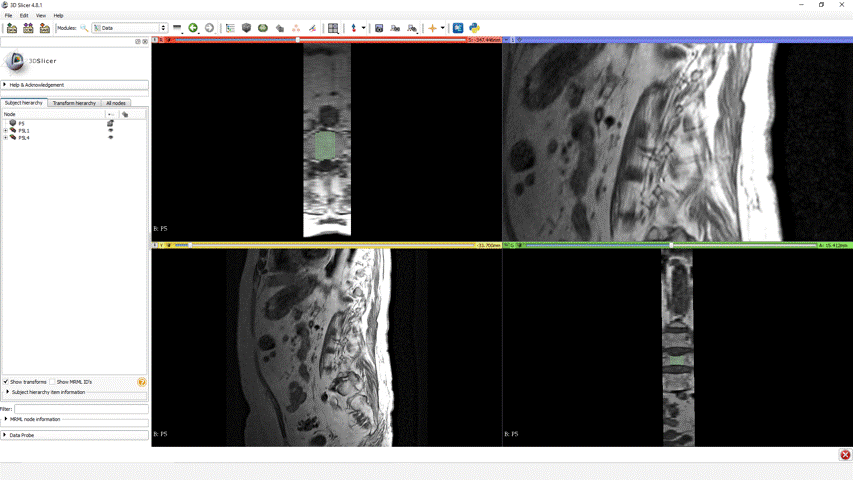


Figure 2 - 3D segmentation over each slice


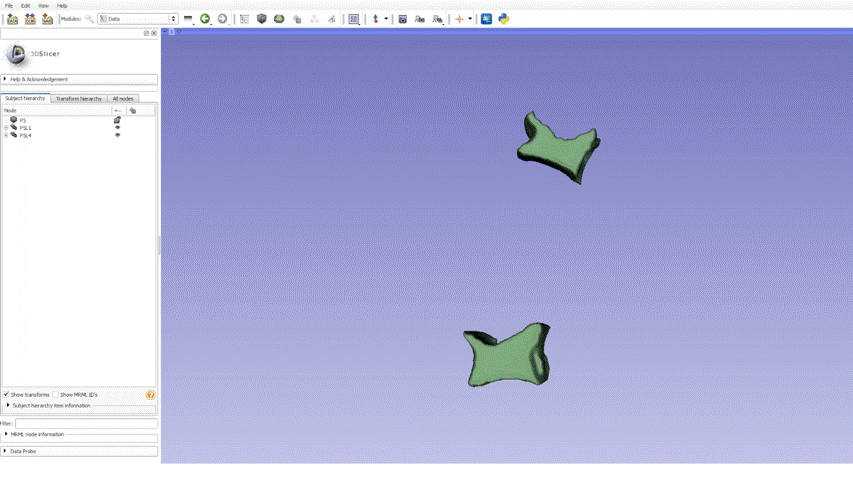


Figure 3 - 3D segmentation

# Extracted Features

| **1** | firstorder_10Percentile | **48** | gldm_GrayLevelVariance |
| --- | --- | --- | --- |
| **2** | firstorder_90Percentile | **49** | gldm_HighGrayLevelEmphasis |
| **3** | firstorder_Energy | **50** | gldm_LargeDependenceEmphasis |
| **4** | firstorder_Entropy | **51** | gldm_LargeDependenceHighGrayLevelEmphasis |
| **5** | firstorder_InterquartileRange | **52** | gldm_LargeDependenceLowGrayLevelEmphasis |
| **6** | firstorder_Kurtosis | **53** | gldm_LowGrayLevelEmphasis |
| **7** | firstorder_Maximum | **54** | gldm_SmallDependenceEmphasis |
| **8** | firstorder_Mean | **55** | gldm_SmallDependenceHighGrayLevelEmphasis |
| **9** | firstorder_MeanAbsoluteDeviation | **56** | gldm_SmallDependenceLowGrayLevelEmphasis |
| **10** | firstorder_Median | **57** | glrlm_GrayLevelNonUniformity |
| **11** | firstorder_Minimum | **58** | glrlm_GrayLevelNonUniformityNormalized |
| **12** | firstorder_Range | **59** | glrlm_GrayLevelVariance |
| **13** | firstorder_RobustMeanAbsoluteDeviation | **60** | glrlm_HighGrayLevelRunEmphasis |
| **14** | firstorder_RootMeanSquared | **61** | glrlm_LongRunEmphasis |
| **15** | firstorder_Skewness | **62** | glrlm_LongRunHighGrayLevelEmphasis |
| **16** | firstorder_StandardDeviation | **63** | glrlm_LongRunLowGrayLevelEmphasis |
| **17** | firstorder_TotalEnergy | **64** | glrlm_LowGrayLevelRunEmphasis |
| **18** | firstorder_Uniformity | **65** | glrlm_RunEntropy |
| **19** | firstorder_Variance | **66** | glrlm_RunLengthNonUniformity |
| **20** | glcm_Autocorrelation | **67** | glrlm_RunLengthNonUniformityNormalized |
| **21** | glcm_ClusterProminence | **68** | glrlm_RunPercentage |
| **22** | glcm_ClusterShade | **69** | glrlm_RunVariance |
| **23** | glcm_ClusterTendency | **70** | glrlm_ShortRunEmphasis |
| **24** | glcm_Contrast | **71** | glrlm_ShortRunHighGrayLevelEmphasis |
| **25** | glcm_Correlation | **72** | glrlm_ShortRunLowGrayLevelEmphasis |
| **26** | glcm_DifferenceAverage | **73** | glszm_GrayLevelNonUniformity |
| **27** | glcm_DifferenceEntropy | **74** | glszm_GrayLevelNonUniformityNormalized |
| **28** | glcm_DifferenceVariance | **75** | glszm_GrayLevelVariance |
| **29** | glcm_Id | **76** | glszm_HighGrayLevelZoneEmphasis |
| **30** | glcm_Idm | **77** | glszm_LargeAreaEmphasis |
| **31** | glcm_Idmn | **78** | glszm_LargeAreaHighGrayLevelEmphasis |
| **32** | glcm_Idn | **79** | glszm_LargeAreaLowGrayLevelEmphasis |
| **33** | glcm_Imc1 | **80** | glszm_LowGrayLevelZoneEmphasis |
| **34** | glcm_Imc2 | **81** | glszm_SizeZoneNonUniformity |
| **35** | glcm_InverseVariance | **82** | glszm_SizeZoneNonUniformityNormalized |
| **36** | glcm_JointAverage | **83** | glszm_SmallAreaEmphasis |
| **37** | glcm_JointEnergy | **84** | glszm_SmallAreaHighGrayLevelEmphasis |
| **38** | glcm_JointEntropy | **85** | glszm_SmallAreaLowGrayLevelEmphasis |
| **39** | glcm_MaximumProbability | **86** | glszm_ZoneEntropy |
| **40** | glcm_SumAverage | **87** | glszm_ZonePercentage |
| **41** | glcm_SumEntropy | **88** | glszm_ZoneVariance |
| **42** | glcm_SumSquares | **89** | ngtdm_Busyness |
| **43** | gldm_DependenceEntropy | **90** | ngtdm_Coarseness |
| **44** | gldm_DependenceNonUniformity | **91** | ngtdm_Complexity |
| **45** | gldm_DependenceNonUniformityNormalized | **92** | ngtdm_Contrast |
| **46** | gldm_DependenceVariance | **93** | ngtdm_Strength |
| **47** | gldm_GrayLevelNonUniformity |  |  |
| Note.― GLCM = gray level cooccurrence matrix. GLDM = gray level dependence matrix. GLSZM = gray level size zone matrix. NGTDM = neighboring gray tone difference matrix. | | | |

The calculation of each feature was performed in accordance with the Image Biomarker Standardization Initiative (IBSI)^[[1]](#footnote-1)^.

1. https://doi.org/10.1148/radiol.2020191145 [↑](#footnote-ref-1)
